# Analysis of the basal chordate *Botryllus schlosseri* reveals a set of genes associated with fertility

**DOI:** 10.1186/1471-2164-15-1183

**Published:** 2014-12-26

**Authors:** Delany Rodriguez, Erin N Sanders, Kelsea Farell, Adam D Langenbacher, Daryl A Taketa, Michelle Rae Hopper, Morgan Kennedy, Andrew Gracey, Anthony W De Tomaso

**Affiliations:** Department of Molecular, Cellular, and Developmental Biology, University of California, Santa Barbara, Santa Barbara, CA 93106 USA; Department of Marine Environmental Biology, University of Southern California, Los Angeles, CA 90089 USA

**Keywords:** Ascidian, Tunicate, Fertility, Infertility, Gonad formation, Germline

## Abstract

**Background:**

Gonad differentiation is an essential function for all sexually reproducing species, and many aspects of these developmental processes are highly conserved among the metazoa. The colonial ascidian, *Botryllus schlosseri* is a chordate model organism which offers two unique traits that can be utilized to characterize the genes underlying germline development: a colonial life history and variable fertility. These properties allow individual genotypes to be isolated at different stages of fertility and gene expression can be characterized comprehensively.

**Results:**

Here we characterized the transcriptome of both fertile and infertile colonies throughout blastogenesis (asexual development) using differential expression analysis. We identified genes (as few as 7 and as many as 647) regulating fertility in *Botryllus* at each stage of blastogenesis. Several of these genes appear to drive gonad maturation, as they are expressed by follicle cells surrounding both testis and oocyte precursors. Spatial and temporal expression of differentially expressed genes was analyzed by *in situ* hybridization, confirming expression in developing gonads.

**Conclusion:**

We have identified several genes expressed in developing and mature gonads in *B. schlosseri*. Analysis of genes upregulated in fertile animals suggests a high level of conservation of the mechanisms regulating fertility between basal chordates and vertebrates.

**Electronic supplementary material:**

The online version of this article (doi:10.1186/1471-2164-15-1183) contains supplementary material, which is available to authorized users.

## Background

**Figure 1:**
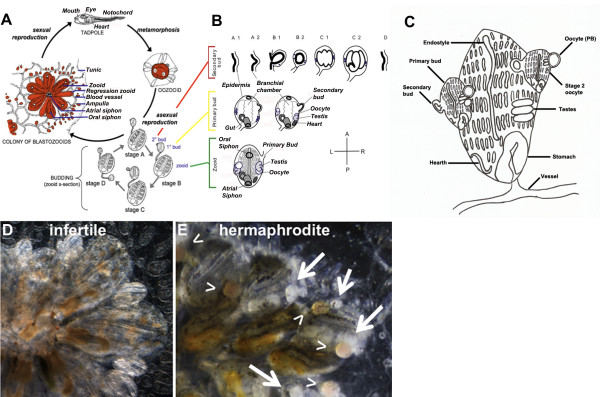
***Botryllus schlosseri*** **sexual and asexual development (blastogenesis) and anatomy. A)** Sexual cycle showing the larva stage and metamorphosis into oozoid. **B)** Asexual cycle – blastogenesis: Dorsal views of zooids (green frame), primary buds (yellow frame) and secondary buds (red frame). A secondary bud appears as a thickening of the epidermis and the peribranchial chamber leaflet of the primary bud (stage A1), which evaginates into a closed vesicle (stage B2), followed by organogenesis (stages C1-D). Gonadogenesis occurs in the secondary bud from mobile precursors (blue; stages B1-C2). During takeover (stages C2-D), the secondary bud becomes the primary bud and a new blastogenic cycle begins for the next secondary bud. After the second takeover event, the primary bud opens its siphons and becomes a functional adult (zooid). In fertile colonies (as illustrated here), the hermaphrodite gonad fully matures on both sides of the zooid. **C)** Schematic showing the anatomy of an adult fertile zooid with two primary buds. **D** and **E)** Ventral live image of both infertile and a fertile colonies (respectively). Arrows point to testes and arrowheads point to eggs. Panels A and B are modified from Brown *et al*. 2009, Laird *et al*. 2005 and Laird and De Tomaso 2005.

*Botryllus schlosseri* is a colonial ascidian found in shallow subtidal marine habitats around the world. Ascidians are urochordates; the sister group of vertebrates [1–4]. Ascidian embryogenesis results in a chordate tadpole larva (that exhibit chordate characteristics such as a notochord, a dorsal nerve cord, pharyngeal slits, and a post-anal tail) that hatches and undergoes a short free-swimming phase. Larvae disperse, find a suitable substrate, then settle and metamorphose (during this process the tail is reabsorbed and the notochord is lost) into the adult form (called an oozooid; Figure 1) that is now sessile. The oozooid has a complex body plan, including a gastrointestinal tract (incurrent and excurrent siphons, pharynx, stomach, intestine), central and peripheral nervous system, an endocrine system, as well as a complex hematopoietic system and extracorporeal vasculature.

*B. schlosseri* belongs to a subset of ascidians that are colonial, and grow not by increasing in size, but by a lifelong, recurring asexual budding process during which entire bodies are regenerated *de novo* every week, resulting in an expanding colony of genetically identical individuals, called blastozooids (hereafter shortened to zooids). Zooids have the same body plan as the oozooid, and arrange themselves into star-shaped structures called systems (Figure 1A). A single tadpole larva can give rise to a colony consisting of thousands of systems with tens of thousands of zooids. The zooids are connected by a common, extracorporeal vasculature that ramifies throughout the colony. However, while linked by a common vasculature, the zooids and buds are independent, and zooids or systems can be separated from the colony and continue to grow (called subcloning). The ability to collect pieces of the same genotype at different time points is a powerful characteristic of *B. schlosseri* as an experimental model for these studies [3].

While the entire colony can have a lifespan ranging from nine months to several years, the zooids themselves are transient. After completing a two-week developmental program, zooids have a defined lifespan of one week as an adult. During that week, new zooids are regenerating in a process called *blastogenesis.* This process is coordinated throughout the colony and arranged spatially such that a colony consists of three co-existing asexual generations within each system (Figure 1A and B) [3–7]. The center of the colony is occupied by the zooids, which are actively feeding and, when fertile, sexually reproducing. They are joined peripherally by primary buds, which are completing development. In turn, these are connected to secondary buds, which are in the initial stages of development. Neither primary nor secondary buds feed or sexually reproduce.

The zooid has a lifespan of one week, then dies in a process called *takeover*. During takeover, all zooids undergo simultaneous apoptosis and are removed by phagocytic cells in the blood. The primary bud then migrates into the newly vacated region of the colony, opens its siphons and becomes a zooid, the secondary bud becomes the primary bud, and a new secondary bud begins developing from a region of the primary bud called the peribranchial epithelium (Figure 1B,C).

### Somatic regeneration

Blastogenesis is synchronized throughout a colony and takes 14 days under laboratory conditions. This process can be divided into distinct visual stages, each lasting one day and demarcated by major morphogenetic changes (illustrated in Figure 1B). As outlined in Figure 1 (panel B), a new generation starts as a secondary bud, first visible as a thickening of the peribranchial epithelium of a primary bud (Stage A1), which evaginates and forms a closed vesicle (Stage B2). Next, a series of epithelial invaginations and protrusions appear as development proceeds (Stage C1). After 7 days (Stage D), a takeover event occurs, the secondary bud transitions to a primary bud, and over the next 7 days completes organogenesis, including initiating new secondary bud(s). At day 14, the zooid of the preceding generation undergoes takeover and is removed, the bud migrates to the new zooid position, and opens its siphons.

### Germline development

Ascidians are hermaphrodites, and adult animals can develop both testes and eggs. Following metamorphosis, colonies undergo an average of eight to twelve developmental cycles prior to the development of gametes (sexual maturity) [8, 9]. In addition, natural populations show seasonal fertility, and in the lab cycle in and out of reproductive (fertile) and non-reproductive (infertile) states [10–13]. In turn, sexuality is also plastic, and colonies can be either hermaphroditic, or male only (with no oocytes visible) (Figure 1D-E)*.*

When a colony is fertile, development of the gametes is synchronized with somatic development [10–13]. Germline stem cells (GSCs) seed the secondary bud (Figure 1B) and begin to proliferate. However, development of oocytes and testis are not linked. Testes develop *in situ*, and are visible in the secondary bud at stage B2, and completing development approximately 10 days later, when that secondary bud becomes a zooid [10–13]. In contrast, oocytes appear to take several cycles to mature (described in detail, below), and oocytes at different stages of development (pre-vitellogenic and vitellogenic) will seed the secondary bud along with the GSCs.

### Egg development

*B. schlosseri* is ovoviviparous and mature eggs are about 300 μm in diameter, and are surrounded by a series of cell layers. These include a layer of outer follicle cells (OFC) a layer of inner follicle cells (IFC) and test cells (TC) [14]. Each egg resembles a small ovary consisting of an oocyte, the envelopes (OFC, IFC and TC) and its own oviduct. Oocyte precursors first appear in secondary buds, ripen in primary buds, and ovulate when the primary buds are transitioning to mature zooids during takeover [14–16]. Oocyte development takes place in the primary bud from stages A1 to C2 and can be described as 5 stages: At stage 1, oocyte precursors are 10 μm in diameter, with a high nuclear to cytoplasmic ratio. At stage 2, the oocyte diameter increases up to 50 μm. At stage 3 oocyte diameter is approximately 80 μ. At stage 4, the oocyte diameter is 120 μm, and vitellogenesis, the process of secretion of nutrients into the oocyte by follicle cells, is initiated. The diameter of stage 5 oocytes is 220 μm, vitellogenesis is almost completed, with yolk granules becoming larger. At the end of the blastogenic cycle one to four oocytes mature and are ovulated into the peribranchial space during takeover (stage D). Usually a number (approximately 6 per primary bud) of oocytes remain arrested at stage 2 (in a previtellogenic stage) which can move from the zooid into the blood stream and reach a new primary bud where they can potentially mature in successive blastogenic generations [3].

To better understand the mechanisms of gonad differentiation and fertility, we used mRNA-seq to identify genes that are differentially expressed between fertile and infertile colonies. Using differential expression analysis combined with *in situ* hybridization, we have identified a number of genes associated with fertility in *Botryllus*, particularly genes expressed by follicle cells - surrounding both testis and oocyte precursors - that may drive maturation. This study is the first step to comprehensively characterize the mechanisms of gonad maturation and fertility in *Botryllus schlosseri*.

## Results and discussion

The recent publication of a draft genome sequence for *Botryllus schlosseri* has identified homologs of vertebrate genes known for their function in lymphoid-mediated immunity as well as eye, ear and heart function [17]. Although this is an important step towards establishing *Botryllus* as a model organism, an analysis of gene expression dynamics throughout the blastogenic cycle is an important tool to further strengthen this non-conventional model system. One of the limitations of a newly established model organism is that we can infer the function of most proteins in *Botryllus* only based on comparison to homologs in other species or analysis of conserved protein domains.

To study spatial and temporal gene expression in *Botryllus*, we combined transcriptomics, quantitative RT-PCR and *in situ* hybridization. To explore the genes involved in gonad formation and fertility, mRNA seq analysis was performed at each stage of the blastogenic cycle (A1, A2, B1, B2, C1, C2 and D) on a total of 3 fertile genotypes and 3 infertile genotypes (Additional file 1: Figure S1 and Additional file 2: Figure S3 and Additional file 3: Table S1, Additional file 4: Table S2, Additional file 5: Table S3, Additional file 6: Table S4, Additional file 7: Table S5, Additional file 8: Table S6, Additional file 9: Table S7). After Quality Control analysis (see Methods), the sequences were mapped to our publicly available *Botryllus schlosseri* EST database Bot_asmb assembly (04.05.2011, A. Gracey) (consisting of 50,107 contigs and representing several genotypes, both fertile but non-pregnant and infertile, at different stages of the blastogenic cycle. See Methods for further details.) (Additional file 10: Table S8 and Additional file 11: Table S9). In order to identify putative homologs of the ESTs in our database, we performed a translated BLAST (blastx) analysis using the non-redundant human protein database (NCBI version 4/25/13) as well as non-redundant protein databases for *Mus musculus, Drosophila melanogaster, Ciona intestinalis and Danio rerio* (an E-value of 1.0 x 10-4 was chosen as the cutoff for a “homolog” for the purpose of this study; Additional file 3: Table S1, Additional file 4: Table S2, Additional file 5: Table S3, Additional file 6: Table S4, Additional file 7: Table S5, Additional file 8: Table S6, Additional file 9: Table S7). Contigs that are differentially expressed (DE) between infertile and fertile animals were detected by DESeq with a false discovery rate (FDR) of 10% (Additional file 3: Table S1, Additional file 4: Table S2, Additional file 5: Table S3, Additional file 6: Table S4, Additional file 7: Table S5, Additional file 8: Table S6, Additional file 9: Table S7, Additional file 12: Table S10 and Additional file 13: Table S11) [18]. Table 1 shows the number of DE contigs at each stage of the blastogenic cycle at a 10% FDR (Additional file 3: Table S1, Additional file 4: Table S2, Additional file 5: Table S3, Additional file 6: Table S4, Additional file 7: Table S5, Additional file 8: Table S6, Additional file 9: Table S7, Additional file 12: Table S10 and Additional file 13: Table S11).

**Table 1:** **Number of differentially expressed EST’s (10% FDR) at each stage of the blastogenic cycle and number of those EST’s with human homologs**

Stage of blastogenesis	Number of differetially expressed genes (10%FDR)	Number of human homologs
A1	592	290
A2	136	70
B1	647	304
B2	125	59
C1	7	2
C2	61	32
D	221	122

Many of the homologs of these contigs have been previously demonstrated to play a role in germline development, fertility and/or fertilization in humans and other organisms (Table 2). In *Botryllus,* conserved genes required for embryonic germ cell specification, such as *vasa*, *piwi* and *nanos,* are expressed in primitive germ cells that are present in juveniles as well as in both fertile and infertile adult animals [19–23]. Therefore, genes that are upregulated in fertile animals in our analysis are likely to be involved in gonad formation/maturation and germ cell differentiation.

**Table 2:** **Differentially expressed genes upregulated in fertile versus infertile colonies and their human and/or ascidian homologs**

DE at Stage(s) of Blastogenesis	EST 5-11 contig name	Homo sapiens or Ascidian homologue	BaseMean Infertile	BaseMean Fertile	FoldChange	pval	padj	Function or Expression	Reference
A1*, A2, B1, C2 and D	CAP3_round1_contig_7511	Histone H1.0	5.33	5031.92	944.26	1.15E-09	3.39E-07	Regulates gene transcription through chromatin remodeling, facilitating expression of fertility-related genes.	[49]
A1, B1*, B2, C1, C2 and D	CAP3_round1_contig_5534	Vitellogenin-1	49.17	45603.55	927.44	2.26E-15	4.49E-12	Involved in secretion of nutrients into the oocyte by follicle cells	[14–16]
A1*, B1, and B2	Bot_c25439	F-box only protein 24 isoform 3	1.18	1062.39	901.21	1.58E-19	1.23E-15	Substrate-recognition component of the SCF (SKP1-CUL1-F-box protein)-type E3 ubiquitin ligase complex	[50]
A1 and B1*	Bot_rep_c36004	Erythrocyte band 7 integral membrane protein isoform a	2.78	1395.43	502.30	4.21E-19	1.64E-15	Expressed on red blood cells.	[51]
A1 and B1*	Bot_rep_c36244	Testis-specific serine/threonine-protein kinase 2	0.72	333.98	462.92	1.59E-15	3.11E-12	Expressed in both mouse and human sperm.	[52]
C2*	Bot_c1378	Microfibrillar-associated protein 4	4.24	1334.72	314.97	1.84E-06	3.61E-03	Involved in photoprotection of the skin.	[53]
A1*, B1 and B2	Bot_c2674	Kelch-like protein 10	3.42	766.95	223.81	5.53E-04	1.41E-01	Encodes a component of intercellular bridges in *Drosophila* egg chambers.	[54]
A1, A2, B1*, and B2	CAP3_round1_contig_5576	Histone H2B type 1-L	3.98	1152.34	289.37	7.74E-15	1.10E-11	Required to condense chromatin in sperm, removes heterochromatin marks, facilitating gene expression	[55]
A1, A2, B1* and B2	Bot_rep_c35370	Creatine kinase S-type mitochondrial	7.80	2193.66	281.17	4.12E-18	1.38E-14	Is expressed in cardiac and striated muscle.	[56]
A1*, B1 and D	Bot_c3513	Tolloid-likeprotein 1	0.61	159.21	261.65	2.42E-08	5.34E-06	Mammalian tolloid (BMP-1) is a proteinase involved in ovarian tissue remodeling	[35]
A1*, A2, B1 and B2	Bot_rep_c45338	Testis-specific serine/threonine-protein kinase 1	6.67	1358.25	203.49	4.31E-16	1.34E-12	Testicular germ cell-specific expression, possible role at and after the meiotic phase of spermatogenesis	[26], 57]
A1, B1, B2*, C2 and D	Bot_rep_c45532	Low density lipoprotein-related protein 1	5.68	1129.89	199.07	3.26E-10	2.18E-06	Mediates endocytosis of cholesterol-rich LDL. In mice, LDLR is expressed in germ cells	[58]
B1, B2 and C2*	Bot_c16824	Zona Pellucida sperm binding protein-1	2.88	568.93	197.42	2.72E-06	4.70E-03	Involved in oocyte development, protection, sperm binding.	[33],59]
A1, A2, B1* and D	Bot_c13044	P-Selectin	1.89	362.01	191.12	9.33E-11	4.21E-08	Expressed in the oolema of oocytes in hamsters and humans, as well as in sperm following the acrosomal reaction	[37–39]
A1 and B1*	CAP3_round1_contig_8583	Ubiquitin-conjugating enzyme E2 R2	10.47	1902.72	181.78	1.04E-11	6.27E-09	Ubiquitin-protein ligase complex, mediates ubiquitination and proteosomal degradation of target proteins during spermatogenesis	[60]
A1 and B1*	Bot_c2928	NAD+-specific isocitrate dehydrogenase beta precursor	7.87	1072.58	136.14	1.78E-05	2.23E-03	Possible role in oxidation of isocitrate to alpha-ketoglutarate in the citric acid cycle.	[61]
A1* and B1	Bot_rep_c45410	Outer dense fiber of sperm tails 3-like 2	5.51	750.03	135.99	3.47E-14	3.69E-11	Structural protein surrounding the axoneme in both the middle piece and principal piece of the sperm tail.	[62]
A1* and B1	Bot_c16543	CD81 antigen (Tetraspanin-8)	1.19	150.88	125.95	2.73E-10	9.77E-08	Expressed by granulosa cells surrounding the oocyte in mice. Reduced fertility in CD81-/- null mice, possible role in acrosomal reaction	[63]
A1*, B1, B2	CAP3_round1_contig_7986	Meltrin-S	5.49	625.35	113.78	1.78E-13	1.74E-10	Mediates cleavage of proteoglycans during the release of the oocyte in mammals	[64]
A1, B1*	CAP3_round1_contig_6892	ADAM metallopeptidase domain 12	35.70	3526.16	98.75	1.55E-09	5.14E-07	ADAMs play roles in spermatogenesis and sperm function, potentially by effecting maturation of sperm and their adhesion and migration	[64, 65]
A1, A2*, and B1	CAP3_round1_contig_4222	Cyclin A1	8.29	808.46	97.45	4.87E-05	3.39E-02	Involved in cell cycle.	[66]
A1 and B1*	Bot_rep_c35625	Serine racemase	14.60	1226.71	84.00	1.37E-13	1.37E-10	Expressed in human testes,pecifically in spermatogonia, spermatocytes, spermatids, Leydig and Sertoli cells	[67]
A1, B1*, B2	CAP3_round1_contig_4558	Fibrous sheath-interacting protein 2	29.98	2389.31	79.67	3.66E-11	1.90E-08	Expressed in late spermatocyte development.	[68]
A1*, B1 and D	Bot_c1197	Hemicentin-1 precursor	22.88	1722.58	75.28	4.39E-13	3.55E-10	Facilitates the gliding of the developing gonad along epithelial basement membranes and germline cellularization.	[69]
A1*, A2 and B1	CAP3_round1_contig_9899	Testis-specific serine/threonine-protein kinase 6	21.59	1245.79	57.70	5.29E-12	3.18E-09	Essential for spem production and function	[70]
A1*, B1 and D	CAP3_round1_contig_9042	OTOA protein	91.00	4925.43	54.12	2.39E-12	1.54E-09	Expressed in sensory epithelia of the inner ear.t Has also been classified as a testis-selective cancer/testis (CT) gene	[27]
A1, A2, B1*, C2 and D	Bot_rep_c50436	Fukutin	21.63	1124.36	51.99	6.28E-06	8.64E-04	Mouse homozygous-null embryos showed folding of the egg cylinder, leakage of maternal red blood cells into the yolk sac cavity.	[71]
A1*, A2 and D	Bot_c2465	ATP-binding cassette sub-family B member 5 isoform 1	59.69	2393.08	40.10	5.75E-11	2.56E-08	In *Drosophila*, ABC transporters efflux prenylated peptides out of somatic gonadal precursors to serve as chemoattractants for migrating germ cells	[72]
A2*	Bot_c25254	Retinol dehydrogenase 12	37.74	1127.87	29.88	2.41E-08	1.87E-04	In *Botryllus schlosseri* retinoic acid signaling is involved in gonad formation	[23]
A1, B1* and D	CAP3_round1_contig_9460	Tubulin beta-4B chain	351.41	10282.63	29.26	1.61E-07	3.41E-05	Tubulin is the major constituent of microtubules; post-translational modification bu monoglycalation specifi incorporation into axonemes.	[73]
A1 and B1*	CAP3_round1_contig_2349	Ciliary dynein heavy chain 9	122.18	3015.52	24.68	1.06E-08	3.02E-06	Possible role in sperm development or motility	[74]
A1*, A2, B1 and D	Bot_rep_c36144	Cyclic nucleotide-gated olfactory channel	46.02	1050.24	22.82	7.82E-09	1.91E-06	Expressed in the flagellum of mature sperm, involved in sperm movement	[75]
A1*	CAP3_round1_contig_7272	Cyclin B3	280.56	1503.78	5.36	5.99E-04	5.22E-02	Involved in cell cycle regulation	[66]

Table 2 includes human and/or ascidian homologs that are differentially expressed (at 10% FDR) throughout the blastogenic cycle. BaseMean Infertile = mean normalized counts from Infertile, BaseMean Fertile = mean normalized counts from Fertile. FoldChange = fold change from Infertile to Fertile, pval = p value for statistical significance; padj = p value adjusted for multiple testing at 10% false discovery rate; Differentially expressed (DE) genes at stage(s) of the blastogenic cycle bold and (*) show the reported basemean infertile and fertile pval, padj for that particular contig at that particular stage.

The number of differentially expressed genes varied greatly between stages of the blastogenic cycle, from a minimum of 7 at stage C1 to a maximum of 647 at stage B1 (Table 1).

In *Botryllus* spermatogenesis begins at stage A1 in the testis of the zooid and continues until stage B2 when mature sperm is released through the oral siphon out into the water [3]. Consistent with this timing, we found that based on homology, a significant portion of the genes upregulated in stages A1 and A2 are functionally related to sperm formation (Additional file 3: Table S1, Additional file 4: Table S2, Additional file 14: Table S12, Additional file 15: Table S13, Additional file 16: Table S14, Additional file 17: Table S15, Additional file 18: Table S16, Additional file 19: Table S17, Additional file 20: Table S18, Additional file 21: Table S19). Since most of these homologs are involved in spermatogenesis in vertebrates (references in Table 2), these data suggest some degree of conservation of the mechanisms governing spermatogenesis between ascidians and vertebrates.

To investigate the spatial and temporal expression pattern of DE genes, we performed quantitative RT-PCR (qPCR) and fluorescent *in situ* hybridization (FISH) for genes that were selected from the most highly DE genes with human homologs: *p-selectin (slep)*, *otoancorin (otoa)*, *tetraspanin-8 (tspan8)*, *low-density lipoprotein receptor (ldlr)*, *testis-specific serine/threonine kinase 1 (tsk1)* and *2 (tsk2*) and genes with known roles in fertility in other organisms: *estradiol 17* β *dehydrogenase-8 (hsd17*β*8), zona pellucida sperm binding protein 1 (zp1), vitellogenin-1 (vtg1), tolloid-like-1 (tll1*). All of these genes were analyzed in fertile colonies at each stage of the blastogenic cycle by qPCR (Additional file 22: Figure S2).

**Figure 2:**
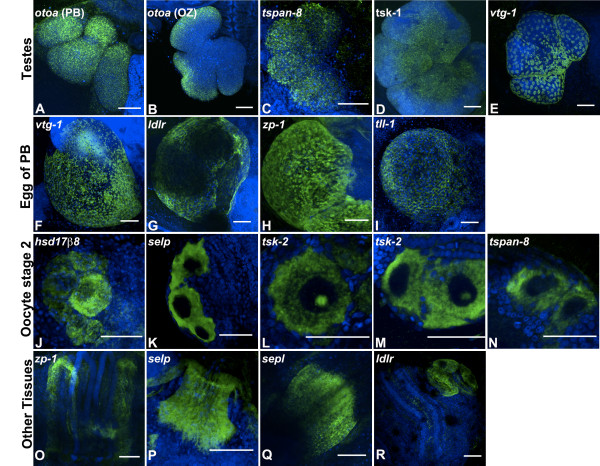
**Fluorescent** ***in situ*** **hybridization (FISH) for selected differentially expressed genes on fertile colonies.** FISH for selected genes with homologs in humans and other organisms was performed on whole mounts of adult fertile colonies. Nuclei were stained with DAPI (blue) and riboprobes are shown in green. Based on their expression patterns in developing gonads, these genes fall into four distinct categories: 1) **(A-E)** localized to testes or developing testes (*otoa, tspan8, tsk1, vtg1*); 2) **(F-I)** localized to maturing eggs (*vtg1, ldlr, zp1, tll1*); 3) **(J-N)** oocytes at stage 2 (*hsd17*β*8, selp, tsk2, tspn8*); and 4) **(O-R)** other tissues (*zp1, selp, and ldlr*). *otoa* = otoancorin, *tspan8* = tetraspanin-8, *tsk1* = testis-specific serine/threonine-protein kinase1, *tsk2* = testis-specific serine/threonine-protein kinase2, *vtg1* = vitellogenin, *ldlr* = Low-density lipoprotein receptor 1, *zp1* = zona-pellucida sperm binding protein 1, *tll1* = Tolloid-like protein1, *hsd17*β*8* = Estradiol 17β dehydrogenase-8, *selp* = P-selectin. Scale bar 50 μm.

We found expression of these genes in: testes or developing testes (*otoancorin, tetraspanin-8, testis-specific serine/threonine kinase 1, vitellogenin*); localized to maturing eggs (*vitellogenin, ldlr, zona-pellucida sperm binding protein-1, tolloid-like protein-1*); oocytes at stage 2 (*estradiol 17*β *dehydrogenase-8, p-selectin, testis-specific serine/threonine kinase 2, tetraspanin-8*); and other tissues (*zona-pellucida sperm binding protein 1, p-selectin, and ldlr*) (Figure 2).

Testis-specific serine/threonine protein kinases phosphorylate myelin basic protein and histones *in vitro*[24]. The testicular germ cell-specific expression of the mouse homologs suggests that these genes play an important role at and after the meiotic phase of spermatogenesis [25]. In *Botryllus, Testis-specific serine/threonine protein kinases*, *tsk-1, tsk-2* and *tsk-6* are upregulated in fertile animals based on our DE analysis (*tsk-1*: 203-fold upregulated at stage A1; *tsk-2*: 462-fold upregulated at stage B1; *tsk-6*: 57-fold upregulated at stage A1). qPCR analysis showed that both *tsk-1* and *tsk-2* are highly upregulated between stages A2 and B2, and *in situ* hybridization indicates that the mRNA is expressed on developing testes (Figure 2) consistent with a crucial role of these genes in spermatogenesis.

In mammals, Otoancorin is specific to sensory epithelia of the inner ear but in humans it has also been classified as a testis-selective cancer/testis gene [26]. Based on our DE analysis, the *Botryllus* homolog of *otoancorin* is upregulated 54-fold in fertile animals at stage A1. By *in situ* hybridization, *otoancorin* shows strong expression from stage A1 through B1 in the testes of the primary bud (pb) (Figure 2A), indicating that in *Botryllu*s, *otoancorin* might be involved in spermatogenesis. By qPCR, its expression level is highest in A1, and decreases as the blastogenic cycle continues. Interestingly, testes of the zooid also show signal for *otoancorin* mRNA, but expression is restricted to the peripheral cells of the testes (Figure 2B).

Tetraspanin/CD9-like is an oocyte factor required for sperm-oocyte fusion [27]. Consistent with these findings in mammals, *in situ* hybridization shows expression of the *Botryllus* homolog of *tetraspanin-8* on stage 2 oocytes. The ADAM-integrin-tetraspanin complex, known to constitute a network of membrane microdomains called the tetraspanin web, is potentially involved in the migration of prespermatogonia from the center to the periphery of the testicular cords and in the reinitiation of mitotic activity during the initial wave of spermatogenesis [28]. By qPCR, expression of *tetraspanin-8* in *Botryllus* is highest at stage A1, coinciding with the initiation of spermatogenesis. Furthermore, our DE analysis revealed a 125-fold upregulation of *tetraspanin-8* in fertile animals at stage A1. *In situ* hybridization showed cells expressing *tetraspanin-8* mRNA in the testes of the primary bud, and suggests that the role of Tetraspanin proteins in spermatogenesis is likely to be conserved between *Botryllus* and vertebrates.

Vitellogenins are lipophosphoglycoproteins that are produced under female hormonal control in large central organs (fat body in insects; liver in higher animals) and are transported in the circulation to the female gonads [29]. In *Botryllus*, a *vitellogenin* homolog is 927-fold upregulated in fertile animals at stage B1. Two novel isoforms of *vitellogenin*, both of which possess vWF-D and CT domains but not a lipovitellin or phosvitin domain, are expressed in the gonad of the ascidian *Halocynthia roretzi*[30]. *In situ* hybridization revealed that mRNAs of these proteins are specifically expressed in oocytes and test cells, accessory cells in the perivitelline space of ascidian eggs. Immunohistochemistry showed that these proteins are localized around the surface of test cells in immature oocytes [30]. In *Botryllus*, *vitellogenin* expression is highest at stage B2 (Additional file 22: Figure S2), and, consistent with the findings in *Halocynthia*, *in situ* hybridization shows expression on maturing eggs of the primary bud (Figure 2).

Interestingly, *vitellogenin* mRNA is also expressed by follicle cells on the testes of both the primary bud and the zooid (Figure 2). These cells are restricted to the periphery of the testes and seem to surround them, in contrast to the mRNA’s of *otoancorin, testis-specific serine/threonine-protein kinase 1 and tetraspanin-8* (Figure 2), which are expressed by cells in the inner compartment of the testes.

To date, all molecularly characterized vitellogenin receptors belong to the low-density lipoprotein receptor (ldlr) supergene family. Ldlr receptors localized in coated pits on the surface of growth-competent oocytes are able to accumulate in the yolk high concentrations of vitellogenin and other ligands they recognize [29]. A *Botryllus schlosseri* homolog of *ldlr* is upregulated 199-fold in fertile animals at stage B2, and qPCR analysis shows that expression of *ldlr* in *Botryllus* is highest at stage A2 (Additional file 22: Figure S2). By *in situ* hybridization, we found that *ldlr* is expressed in follicle cells of maturing eggs in the primary bud (Figure 2), suggesting that it might function as a vitellogenin-receptor during oocyte maturation.

In *Halocynthia roretzi*, a 120-kDa transmembrane protein with a zona pellucida domain and a 13 EGF-like repeats (HrVC120), has been shown to be the receptor in the vitelline coat that binds to sperm [31]. *Ciona intestinalis* has several genes related to HrVC120 that are exclusively expressed in developing oocytes but not in eggs [32]. We found a gene containing a Zona Pellucida domain, *zp-1*, that is highly upregulated in fertile animals (197-fold change at stage C2) and highly expressed at stage A1 (Additional file 22: Figure S2). By *in situ* hybridization, we found that *zp-1* is expressed by the follicle cells that surround the egg in the primary bud. To our knowledge this is the first report of a gene with a Zona Pellucida domain in a colonial ascidian. This finding reflects the close relationship of these animals with vertebrates and the fact that this domain has been maintained in deuterostomes suggests that it is indispensable for sperm-egg interaction [27, 33].

Mammalian *Tolloid* (*Bmp-1*) is a proteinase involved in ovulation. During ovarian tissue remodeling, it contributes to the maturation of procollagen molecules and the deposition of collagen fibrils [34]. In *Botryllus*, a *tolloid-like protein 1* homolog is expressed on follicle cells of maturing oocytes of the primary bud and is upregulated 261-fold in fertile animals at stage A1 (Figure 2).

A *Botryllus schlosseri* homolog of *estradiol 17* β *dehydrogenase-8* (*hsd17*β*8*) is highly upregulated in fertile animals (5.4-fold at stage B1), and as expected, given its involvement in estrogen metabolism [35], its mRNA localizes to oocytes at stage 2 (Figure 2).

A *Botryllus schlosseri* homolog of *p-selectin* (selp) is upregulated 191-fold in fertile animals at stage B1. By *in situ* hybridization, this gene is expressed on several stages of oocytes (Figure 2). The function of this protein in germ cells is poorly understood, but it is expressed in the oolema of oocytes in hamsters and humans, as well as in sperm following the acrosomal reaction, and is hypothesized to be involved in sperm-oocyte adhesion [36–38]. We measured the diameter of the oocytes expressing *p-selectin*, and found that their size increased as the blastogenic cycle advanced (Table 3). Cells positive for *p-selectin* mRNA range from 16–21 μm in diameter at stage A1, suggesting they are stage 2 oocytes as classified by Manni [3, 14–16]. Stage 2 oocytes are defined as being greater than 10 μm in diameter (bigger than stage 1 oocyte precursors) but less than 50 μm in diameter (smaller than stage 3 oocytes). By the middle of the blastogenic cycle, *p-selectin*-positive cells can reach up to 39 μm (stage B2). By the end of blastogenesis, one or two *p-selectin*-positive cells per primary bud will be about 42 μm in diameter, and could indicate that these cells are committed to become mature oocytes [14–16]. This supports the concept that there is a persisting reservoir of egg-precursors, and only one or two of those cells will increase in size to move on to stage 3 and develop into the egg of the primary bud for the following blastogenic cycle [14–16].

**Table 3:** **Measurements of** ***p-selectin*** **positive cells, mean and standard deviation (SD) in the primary bud throughout the blastogenic cycle**

Stage	Range of diameters ( μm)	Mean ( μm)	SD ( μm)
A1	16-21	26.35	4.14
A2	20-30	20.42	2.98
B1	25-37	35.58	5.46
B2	23-39	29.76	8.44
C1	22-35	31.35	5.59
C2	19-42	28.48	5.82
D	15-22	18.18	7.33

Unexpectedly, we found that stage 2 oocytes were positive for the mRNA of *testis-specific serine/threonine-protein kinase 2* gene at stage B1 and B2 of the blastogenic cycle. Moreover, a strong nucleolus like signal was observed in about 30-40% of positive cells. This finding could indicate a previously unknown function of this gene in oogenesis.

**Figure 3:**
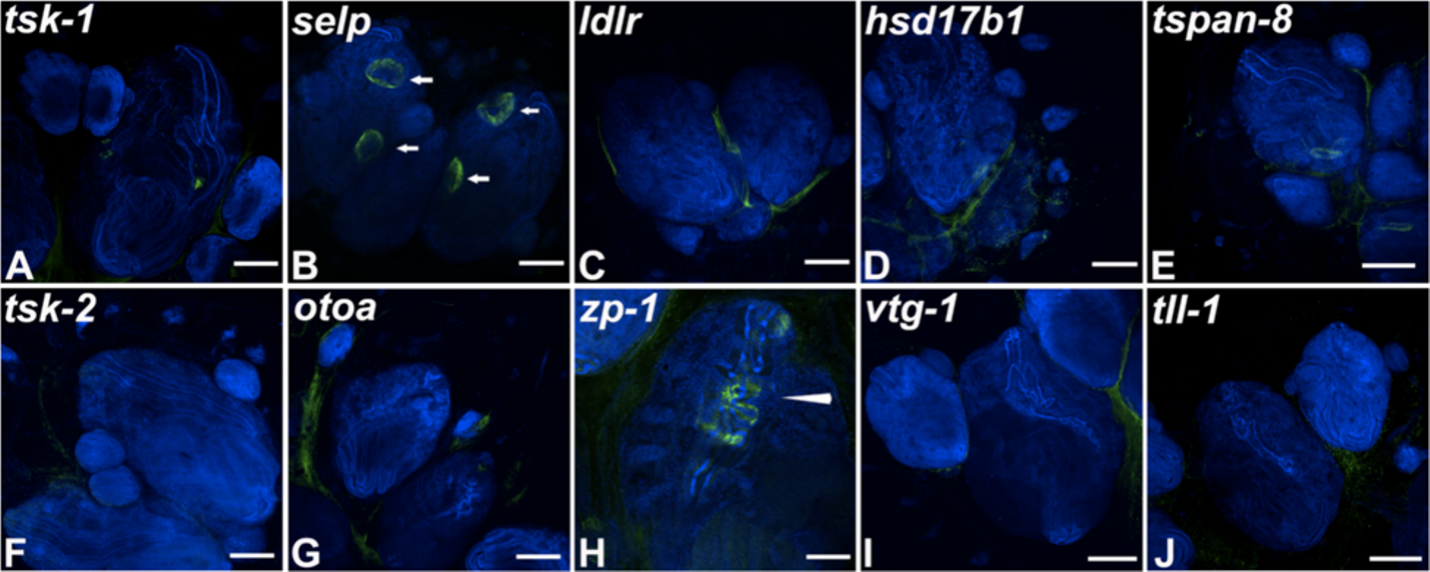
**Fluorescent** ***in situ*** **hybridization (FISH) for selected differentially expressed genes on infertile colonies.** FISH for selected genes was performed on whole mounts of adult infertile colonies **(A-J)**. Nuclei were stained with DAPI (blue) and riboprobes are shown in green. Expression of genes detected in fertile colonies is absent in juveniles except for p-selectin (localizing to both oral and excurrent siphons) as indicated by arrows **(B)** and Zona-pellucida sperm binding protein1 (localizing to the endostyle) as indicated by the arrowhead **(H)**. *otoa* = otoancorin, *tspan8* = tetraspanin-8, *tsk1* = testis-specific serine/threonine-protein kinase1, *tsk2* = testis-specific serine/threonine-protein kinase2, *vtg1* = vitellogenin, *ldlr* = Low-density lipoprotein receptor 1, *zp1* = zona-pellucida sperm binding protein 1, *tll1* = Tolloid-like protein1, *hsd17b8* = Estradiol 17β dehydrogenase-8, *selp* = P-selectin. Scale bar 200 μm.

Finally, we observed that some of the genes upregulated in fertile animals are expressed in tissues outside of the germ line. Particularly *p-selectin* mRNA is expressed on the oral and excurrent siphons of both the zooid and primary bud (Figure 3B arrows). Interestingly, at stage D of the blastogenic cycle, *p-selectin* positive cells are arranged in a calyx shape (Figure 2Q) of the primary bud as it transitions to a zooid during takeover. A *Botryllus schlosseri* homolog of *Zona pellucida binding protein-1* showed mRNA expression on cells that run along the endostyle of both zooid and primary bud, while *ldlr* showed positive mRNA expression on secondary bud tissue at stage B1.

The set of fertility-related genes we have identified represents the first reported molecular markers for a variety of *Botryllus schlosseri* anatomical structures. Specifically, we found transcripts expressed by the testes (both developing and mature testes), by follicle cells surrounding both maturing oocytes and testes, by oocytes at stage 2 and by the siphons (oral and excurrent). These markers will undoubtedly serve as a valuable resource for other researchers examining fertility in *Botryllus*.

To obtain a broader perspective and explore the biological roles of the genes identified in our DE analysis, we performed a gene ontology (GO) analysis on the human homologs of differentially expressed genes (Table 1) at each stage of the blastogenic cycle (Human genes were used as a proxy in our GO analysis because *Botryllus* ESTs lack GO annotation) [39–41]. Specifically, annotation based on biological processes provided significant insight into functional changes related to fertility throughout the blastogenic cycle, and are discussed here. Other GO annotations (All annotations and molecular function) are included in Additional file 14: Table S12, Additional file 15: Table S13, Additional file 16: Table S14, Additional file 17: Table S15, Additional file 18: Table S16, Additional file 19: Table S17, Additional file 20: Table S18, Additional file 21: Table S19, Additional file 23: Table S20, Additional file 24: Table S21, Additional file 25: Table S22, Additional file 26: Table S23, Additional file 27: Table S24, Additional file 28: Table S25, Additional file 29: Table S26, Additional file 30: Table S27, Additional file 31: Table S28, Additional file 32: Table S29, Additional file 33: Table S30, Additional file 34: Table S31, Additional file 35: Table S32, Additional file 36: Table S33, Additional file 37: Table S34, Additional file 38: Table S35, Additional file 39: Table S36, Additional file 40: Table S37 and Additional file 41: Table S38. Furthermore, we conducted Inter Pro analysis to find conserved protein domains (Additional file 17: Table S15, Additional file 21: Table S19, Additional file 26: Table S23, Additional file 30: Table S27, Additional file 37: Table S34, and Additional file 41: Table S38).

At stages A1 and A2, 592 (290 with human homologs) and 136 (70 with human homologs) genes are upregulated in fertile animals, respectively. GO analysis revealed that genes related to male meiosis, spermatogenesis, cilium, proteolysis, microtubule based movement, meiosis, spermatid development, cilliary or flagellar motility, female gamete generation and male meiosis are upregulated (Additional file 14: Table S12, Additional file 15: Table S13, Additional file 16: Table S14, Additional file 18: Table S16, Additional file 19: Table S17 and Additional file 24: Table S18), correlating with the fact that spermatogenesis begins in this timeframe. Inter Pro analysis revealed that these genes contain domains such as EGF, LDLR, BMP, Selectin and Meiotic recombination Spo11, among others (Additional file 21: Table S19 and Additional file 26: Table S23). These results are congruent with previous non-molecular observations indicating that gametogenesis starts during these stages.

At stages B1 and B2, 647 and 125 genes are DE with 304 and 59 human homologs respectively. Genes related to male meiosis, spermatogenesis, spermatid development, microtubule-based movement, cell differentiation, motility, filipodium assembly, flagellar motility, cell adhesion, female gamete generation and apoliprotein binding are upregulated in fertile animals (Additional file 23: Table S20, Additional file 24: Table S21, Additional file 25: Table S22, Additional file 27, Table S24, Additional file 28: Table S25 and Additional file 29: Table S26). Inter Pro analysis revealed that these genes contain domains such as EGF, Kringle, CUB, Speract/Scavenger Kelch, Calcium binding, ADAM, and Peptidase S1/S6/M12A, BMP1/Tolloid, Zona pellucida sperm-binding protein and LDLR among others (Additional file 26: Table S23 and Additional file 30: Table S27). These results reflect the complex biological processes occurring during these stages, which also have the highest number of DE genes, and are consistent with the timing of sperm maturation and release.

At stage C1 and C2, 7 and 61 genes are DE with 2 and 32 human homologs respectively. The predominant GO terms at these stages included oocyte maturation, oocyte growth, lipoprotein metabolic process, regulation of cholesterol transport, positive regulation of estrogen receptor, extracellular matrix disassembly, and tissue remodeling, among others (Additional file 31: Table S28, Additional file 32: Table S29, Additional file 34: Table S31, Additional file 35: Table S32 and Additional file 36: Table S33). The Inter Pro analysis for this stage revealed domains such as: Apple-like, LDLR, Kringe, Aquaporin, Tetraspanin, EGF, Zona Pellucida sperm-binding protein, Peptidase S1/S6/M12A and FY rich, among others (Additional file 33: Table S30 and Additional file 37: Table S34). At these stages maturing oocytes rapidly increase in size and are preparing to move into the branchial basket of the zooid.

Finally at stage D 221 genes are DE (with 122 human homologs). GO analysis revealed the presence of annotations including cell adhesion, carbohydrate metabolism, regulation of developmental process and response to corticosteroids stimulus, proteolysis, lipoprotein transport, cholesterol transport, estrogen receptor signaling pathway, oocyte growth (Additional file 38: Table S35, Additional file 39: Table S36 and Additional file 40: Table S37) and the Inter Pro analysis of the genes upregulated at this stage revealed the following protein domains: EGF, Notch, CUB, LDLR, Selectin, and Peptidases S1/S1A/S6, among others (Additional file 41: Table S38). At this stage mature oocytes begin to transition from the peribranchial cavity into the branchial basket of the new zooid.

In summary, our GO analysis has revealed a number of genes with potential involvement in spermatogenesis at stages A1-B2, correlating with what has been described for *Botryllus schlosseri*[3]. The *Botryllus* homologs of these genes are promising candidates for future studies on testes maturation and sperm production. Homologs of genes with reported roles in lipid and steroid metabolism are upregulated at stages B1-D, representing key processes involved in oocyte maturation in other species that may be conserved in *Botryllus*[42–44].

## Conclusions

This manuscript represents the first step in the comprehensive characterization of gonad differentiation and fertility in *Botryllus schlosseri*. We have analyzed the transcriptomes of both infertile and fertile colonies and characterized the spatial and temporal expression of highly expressed genes involved in fertility. Our findings suggest a high level of conservation of the mechanisms regulating fertility between basal chordates and vertebrates. Some of the genes characterized in this study were found to have unexpected spatial localization, such as *testis serine/threonine kinase 2*, which is expressed by egg precursors. Another example is *vitellogenin*, which localizes to both follicle cells surrounding the egg and equivalent cells surrounding the testes.

Interestingly, known germline markers such as *vasa*, *nanos* and *piwi* were not significantly differentially expressed between fertile and infertile colonies, most likely because both juveniles and infertile colonies have immature germ cells expressing these genes. We have identified specific markers for both developing gonads (and mature testes) throughout the blastogenic cycle in zooids, primary buds and secondary buds. Some of these markers are now being used to investigate the phenomenon of induced juvenile fertility in *Botryllus schlosseri* (Di Maio *et al.* unpublished).

In future studies, we will investigate differential gene expression between male and hermaphroditic colonies, which will allow us to characterize genes used exclusively in male or female gamete development and identify genes governing the process by which *Botryllus schlosseri* assumes a given reproductive state.

## Methods

### Animals

All mariculture procedures have been described previously (Boyd *et al.*, 1986). Briefly, we collected pregnant *Botryllus schlosseri* colonies from the Santa Barbara Marina, California. Individual colonies were tied to 3 × 5-cm glass slides and placed 5 cm opposite another glass slide (called the settlement slide) in a slide rack. The slide rack was placed into an aquarium, and within a few days the tadpoles hatched, swam to the settlement slide, and metamorphosed into the adult body plan (oozooid). Over 95% of the tadpoles hatch and immediately swim to and settle on the juxtaposed slide [45]. Animals are reared in 5 L tanks supplemented with food in suspension daily, and food is not limiting [45]. Only isolated genotypes reared in our mariculture facility were used for this study.

To explore only the genes involved in gonad formation and fertility, mRNA seq analysis was performed at every stage of the blastogenic cycle (A1, A2, B1, B2, C1, C2 and D, each stage as described by bud morphogenesis based on *in vivo* and histological features [46]) on a total of 3 fertile samples (SB801, SB841 and Mix genotypes SB802a, SB804, SB722 ) and 3 infertile samples (SB802d, SB-BB-001 and Mix genotypes SB831, SB645, SB714, SB841, SB842 ).

### RT-PCR, cloning, and quantitative PCR

Total RNA was isolated from whole colonies using Nucleo-Spin II columns from Macherey Nagel, and mRNA was isolated using the NEB Magnetic Bead Isolation kit. cDNA was made using Superscript II from Invitrogen. For RT-PCR, we used Clontech Advantage following the manufacturers′ recommendations for PCR conditions.

PCR products were isolated using Qiagen columns, then cloned into the Promega Easy-T vector and transformed using competent NEB5α from NEB. Single colonies were picked and inserts amplified using colony PCR and Clontech Advantage. PCR products were prepared for sequencing then sent for Sanger sequencing at the UC Berkeley Sequencing Facility.

Quantitative PCR (QPCR) analysis was done as described previously [47]. Briefly, Q-PCR was carried out using a LightCycler 480 II (Roche) and LightCycler DNA Master SYBR Green I detection (Roche, 12015099001). The thermocycling profile was as follows: 5 minutes at 95°C, 45 cycles of 95°C for 10 sec, 50-60°C for 10 sec, and 72°C for 10 sec. All gene expression data was normalized to *elongation factor 1*-α (*EF1-* α) as a reference house keeping gene and reported as relative expression using the 2^-ΔΔCt^ method.

Three biological and technical replicates were used for each gene.

Primers for QPCR are shown in Table 4.

**Table 4:** **Primers sequence for QPCR**

Gene name	Primers sequence 5′-3′
*tll-1*	Fwd-TTGCTGCACATCGAGTGTCT
	Rev-GTGCTACAAGTTGCGAGTGC
*tsk-2*	Fwd-GGCGTGGATTACCTTCACGA
	Rev-AAGCTCCATACGTCGGCAAT
*ldlr*	Fwd-GTGGTTCTGGAACCGGATGT
	Rev-TGACGAGTGCTCATCGGAAC
*tspan-8*	Fwd-AGTGGGAATTTGGGCTCTCG
	Rev-AGTCACAAGCGTCATCGGAG
*tsk-1*	Fwd-TTCGAAACACTCTGGGCGAA
	Rev-TCGGATGACTCAAGAGGGGT
*vtg-1*	Fwd-CGAGTCATGTATGTCCGCGA
	Rev-TGAAAGGCGAGCTGCAGTAA
*zp-1*	Fwd-GCAACGATCCATTTCCGTCG
	Rev-CTCGTCCAATGTGGCAGAGT
*otoa*	Fwd-AGATTTCGAGTTGCTTTTACAGGTTAC
	Rev-CAATACTTCAAAGACTGTTTCCTCTGT
*hsd17β8*	Fwd-GCGGTCTGGTAAGCTTTGGT
	Rev-GTCGGACGCAAGGAACAAAC
*selp*	Fwd-CTATTCTCTATACCCCGAAGGCTTTAC
	Rev-CTTCAGAGATTGTGAAAGAAGCAAT

### mRNA seq

Total RNA was isolated from whole colonies using the Nucleo-Spin RNA II kit (MN). Libraries were prepared and sequenced at the USC Epigenome Center using kits from Illumina following the manufacturers’ instructions. Paired-end libraries were generated for each sample and sequenced with an Illumina Hi-Seq 2000. The data supporting the results of this article have been deposited in NCBI’s Gene Expression Omnibus [48, 49] and are accessible through GEO Series accession number GSE62112 (http://www.ncbi.nlm.nih.gov/geo/query/acc.cgi?acc=GSE62112). After RNASeq each paired end (PE) library was first checked for quality control (QC) using the software FastQC (http://www.bioinformatics.babraham.ac.uk/projects/fastqc/ ) with an average score of 28 across all bases (Illumina 1.5 encoding). Using FASTQ Trimmer we removed the adapters of the sequences by removing the first 12 bases of each read. All reads pairs passing QC were mapped using BOWTIE (2012 version 0.12.7) [50] to the public EST database Bot_asmb assembly (04.05.2011, A. Gracey) (http://octopus.obs-vlfr.fr/public/botryllus/blast_botryllus.php). Bot_asmb assembly (04.05.2011, A. Gracey), consisting of 50,107 contigs, is an assembly of two cDNA libraries generated from a mixture of *Botryllus schlosseri* genotypes at each stage of the blastogenic cycle and representing both infertile and fertile states. The libraries used to construct Bot_asmb assembly (04.05.2011, A. Gracey) consist of: (1) 10,000 arrayed clones sequenced from 5’ to 3’ ends using the Sanger method (200,000 sequences total with an average length of 950 base pairs) and (2) 454 sequencing of the same RNA with an average length of 400 base pairs. The number of reads mapping to each EST were obtained with Sam2Counts (Samtools version 0.1.18) and differential expression analysis was performed with DESeq 1.10.1 using triplicates for the analysis and a false discovery rate of 10% [18]. Fragments per kilobase per million (FPKM) were calculated using RSEM 1.2.3 for each paired-end library for each genotype at each stage of the blastogenic cycle for both infertile and fertile colonies. To identify putative homologs we blasted (BLASTX) to the non-redundant protein databases of *Homo sapiens Mus musculus*, *Drosophila melanogaster*, *Ciona intestinalis* and *Danio reirio* with an E-value cutoff of 1 × 10^-4^.

All bioinformatics analysis was performed on a Desktop computer running Ubuntu 12.10 and equipped with 16 GB of RAM.

### Gene Ontology and InterPro analysis

The list of human homologs of differentially expressed *Botryllus* contigs was submitted to GeneCodis 3, an on-line modular enrichment tool [39–41]. For this analysis, the following annotations were selected: GO Biological Process, GO Molecular Function and InterPro Motifs. The statistical parameters for these analyses were as follows: First, for co-occurrence analyses of annotations, a minimum support of 3 genes was required. Second, the statistical method to compute p-values was the hypergeometric test. Finally, to correct p-values for multiple hypothesis testing, FDR estimation was utilized. Of particular interest to this study were processes and pathways with a known role in fertility and gametogenesis.

### Fluorescent *in situ*hybridization (FISH)

Fragments of *Botryllus schlosseri* Estradiol *17-*β*-dehydrogenase 8* (*hsd17b8*), *P-Selectin* (*selp*), *Otoancorin* (*otoa*), *Testis-specific serine/threonine-protein kinase 2* (*tsk2*)*, Vitellogenin* (*vtg1*), *Tetraspanin 8 (tspan8), Zona-pellucida sperm binding protein 1* (*zp1*), *Low-Density Lipoprotein receptor* (*ldlr*), *Testis-specific serine/threonine-protein kinase 1* (*tsk1*)*, Tolliod-like 1* (*tll1*), ranging in size from 200 bp – 1 kb were amplified with Advantage cDNA polymerase (Clontech) and cloned into the pGEM-T Easy vector (Promega). SP6 or T7 RNA polymerase (Roche) were used to generate antisense RNA probes labeled with digoxigenin (Roche) or DNP (Perkin Elmer).

Single systems of fertile or infertile *Botryllus* were fixed with 4% formaldehyde in 0.1 M MOPS pH 7.5, 0.5 M NaCl for 3 hours and then transferred to methanol. To eliminate pigmentation, animals were bleached in 6% H_2_O_2_/methanol and then stored in methanol at -20°C. After rehydration, animals were permeabilized with proteinase K (10 μg/ml for 30 min) and then post-fixed with 4% formaldehyde. Prehybridization was carried out for 4 hours at 65°C in hybridization buffer (65% formamide, 5X SSC, 1X Denhardt's solution, 0.1% Tween-20, 5 mg/ml torula yeast RNA, 50 μg/ml heparin), followed by hybridization with DIG- and DNP-labeled probes in hybridization buffer overnight at 65°C. After washing off unbound probes, DIG-labeled probe was detected with an anti-DIG HRP-conjugated antibody (Roche) and TSA Plus fluorescent tyramide conjugate (Perkin Elmer). For double labeling, the HRP-conjugated anti-DIG antibody was then inactivated with 2% H_2_O_2_ in PBS with 0.1% Triton X-100 and DNP-labeled probes were detected with anti-DNP HRP-conjugated antibody (Perkin Elmer) and TSA Plus fluorescent tyramide conjugate (Perkin Elmer). After washing in PBS, specimens were flat-mounted with Vectashield (Vector Labs) and imaged on an Olympus Fluoview 1000 Spectral confocal microscope equipped with a 40X oil immersion objective. All FISH experiments were performed in triplicates. Only consistent reproducible staining throughout the whole-mount colonies was reported.

Primers for *in situ* hybridization probes are shown in Table 5.

**Table 5:** **Primer sequences for** ***in situ*** **hybridization probes**

Gene symbol	Primers sequence used for each gene 5′-3′
*hsd17β8*	Fwd-GCGGTCTGGTAAGCTTTGGT
	Rev-GTCGGACGCAAGGAACAAAC
*selp*	Fwd-CCTTCAGTTGCAACAAGGGC
	Rev-ACCGTTTCGGAGAGTTTCCC
*tsk-2*	Fwd-AAAATGCGAGAACATCCTACTCA
	Rev-TTCTGTTAGGTCCCCTGATTGTA
*otoa*	Fwd-CGCAGCGCAGGATTAAACTC
	Rev-GCCGTATTTGTCGTGGATGC
*vtg-1*	Fwd-CCGAACCGTACGGATACCTG
	Rev-TACGTTTTGGACGAAGGCGA
*tspan-8*	Fwd-CAATTTGGCGCTGTTCCTCG
	Rev-CACGACTCTTACGCGTATCCA
*zp-1*	Fwd-AGACGTGGTACCCATAGCCT
	Rev-ACGCTTATCGTGCAAGTGGA
*ldlr*	Fwd-GAATTTGCAGCTCGCTCTCG
	Rev-CATGACGAGTGCTCATCGGA
*tsk-1*	Fwd-GGAAAATGCTCGGAACGGTG
	Rev-CCCGAAAAACGGATGTCCA
*tll-1*	Fwd-TTGCTGCACATCGAGTGTCT
	Rev-TGCCGAAGAAAACGCTGTTG

### Measurement of stage 2 oocytes

After FISH (above) the diameter of *selp* positive cells were measured using FIJI image processing software. Each primary bud (average of five buds per experiment) with positive cells (average of ten positive oocytes stage 2) were measured of total of three colonies at each stage of the blastogenic cycle. Standard deviation was calculated using Microsoft Office Excel 2011.

## Electronic supplementary material

Additional file 1: **Figure S1.** Scatter plots of Differentially Expressed genes throughout the entire blastogenic cycle of *Botryllus schlosseri*. Plot of normalized mean versus log2 fold change infertile versus fertile for each stage of the blastogenic cycle. Red circles indicate genes that are significant at a 10% false discovery rate (FDR). (PDF 615 KB)

Additional file 2: Figure S3: Heat Maps of Differentially expressed genes of both infertile and fertile colonies at each stage of the blastogenic cycle of *Botryllus schlosseri*. Aqua color indicates relative low abundance/expression and darker blue indicates higher abundance/expression. (TIFF 14 MB)

Additional file 3: Table S1: Differentially Expressed genes between infertile and fertile colonies of *Botryllus schlosseri* at stage A1: baseMean = mean normalized counts average over all samples from both conditions, baseMean Infertile = mean normalized counts from Infertile samples, baseMean Fertile = mean normalized counts from Fertile samples, foldChange = fold change from Infertile to Fertile, log2FoldChange = the logarithm (to basis 2) of fold change, pval = p value for the statistical significance, padj = p value adjusted for multiple testing with the Benjamini-Hochberg procedure. (XLS 14 MB)

Additional file 4: Table S2: Differentially Expressed genes between infertile and fertile colonies at stage of *Botryllus schlosseri* A2: baseMean = mean normalized counts average over all samples from both conditions, baseMean Infertile = mean normalized counts from Infertile samples, baseMean Fertile = mean normalized counts from Fertile samples, foldChange = fold change from Infertile to Fertile, log2FoldChange = the logarithm (to basis 2) of fold change, pval = p value for the statistical significance, padj = p value adjusted for multiple testing with the Benjamini-Hochberg procedure. (XLS 14 MB)

Additional file 5: Table S3: Differentially Expressed genes between infertile and fertile colonies of *Botryllus schlosseri* at stage B1: baseMean = mean normalized counts average over all samples from both conditions, baseMean Infertile = mean normalized counts from Infertile samples, baseMean Fertile = mean normalized counts from Fertile samples, foldChange = fold change from Infertile to Fertile, log2FoldChange = the logarithm (to basis 2) of fold change, pval = p value for the statistical significance, padj = p value adjusted for multiple testing with the Benjamini-Hochberg procedure. (XLS 14 MB)

Additional file 6: Table S4: Differentially Expressed genes between infertile and fertile colonies of *Botryllus schlosseri* at stage B2: baseMean = mean normalized counts average over all samples from both conditions, baseMean Infertile = mean normalized counts from Infertile samples, baseMean Fertile = mean normalized counts from Fertile samples, foldChange = fold change from Infertile to Fertile, log2FoldChange = the logarithm (to basis 2) of fold change, pval = p value for the statistical significance, padj = p value adjusted for multiple testing with the Benjamini-Hochberg procedure. (XLS 14 MB)

Additional file 7: Table S5: Differentially Expressed genes between infertile and fertile colonies of *Botryllus schlosseri* at stage C1: baseMean = mean normalized counts average over all samples from both conditions, baseMean Infertile = mean normalized counts from Infertile samples, baseMean Fertile = mean normalized counts from Fertile samples, foldChange = fold change from Infertile to Fertile, log2FoldChange = the logarithm (to basis 2) of fold change, pval = p value for the statistical significance, padj = p value adjusted for multiple testing with the Benjamini-Hochberg procedure. (XLS 14 MB)

Additional file 8: Table S6: Differentially Expressed genes between infertile and fertile colonies of *Botryllus schlosseri* at stage C2: baseMean = mean normalized counts average over all samples from both conditions, baseMean Infertile = mean normalized counts from Infertile samples, baseMean Fertile = mean normalized counts from Fertile samples, foldChange = fold change from Infertile to Fertile, log2FoldChange = the logarithm (to basis 2) of fold change, pval = p value for the statistical significance, padj = p value adjusted for multiple testing with the Benjamini-Hochberg procedure. (XLS 10 KB)

Additional file 9: Table S7: Differentially Expressed genes between infertile and fertile colonies of *Botryllus schlosseri* at stage D: baseMean = mean normalized counts average over all samples from both conditions, baseMean Infertile = mean normalized counts from Infertile samples, baseMean Fertile = mean normalized counts from Fertile samples, foldChange = fold change from Infertile to Fertile, log2FoldChange = the logarithm (to basis 2) of fold change, pval = p value for the statistical significance, padj = p value adjusted for multiple testing with the Benjamini-Hochberg procedure. (XLS 46 KB)

Additional file 10: Table S8: Mapped reads to EST 5–11 for Fertile Samples. (XLSX 11 MB)

Additional file 11: Table S9: Mapped reads to EST 5–11 for Infertile Samples. (XLSX 11 MB)

Additional file 12: Table S10: FPKM for each stage of the blastogenic cycle for Fertile Samples. (XLS 78 KB)

Additional file 13: Table S11: FPKM for each stage of the blastogenic cycle for Infertile Samples. (XLS 37 KB)

Additional file 14: Table S12: Gene Ontology analysis (All annotations) of human homologs of differentially expressed genes at stage A1. Items = Codes of annotations, Items Details = description of annotations, Support = number of genes in input list with a given annotation, List size = number of genes in input list, Reference Support = number of genes in reference list with a given annotation, Reference size = number of genes in reference list, Hyp = Hypergeometric p-value, Hyp c = corrected Hypergeometric p-value (FDR), Genes = genes with given annotation in the input list. (XLS 36 KB)

Additional file 15: Table S13: Gene Ontology analysis of Biological Processes of human homologs of differentially expressed genes at stage A1. Items = Codes of annotations, Items Details = description of annotations, Support = number of genes in input list with a given annotation, List size = number of genes in input list, Reference Support = number of genes in reference list with a given annotation, Reference size = number of genes in reference list, Hyp = Hypergeometric p-value, Hyp c = corrected Hypergeometric p-value (FDR), Genes = genes with given annotation in the input list. (XLS 47 KB)

Additional file 16: Table S14: Gene Ontology analysis of Molecular Function of human homologs of differentially expressed genes at stage A1. Items = Codes of annotations, Items Details = description of annotations, Support = number of genes in input list with a given annotation, List size = number of genes in input list, Reference Support = number of genes in reference list with a given annotation, Reference size = number of genes in reference list, Hyp = Hypergeometric p-value, Hyp c = corrected Hypergeometric p-value (FDR), Genes = genes with given annotation in the input list. (XLS 46 KB)

Additional file 17: Table S15: Inter Pro analysis of human homologs of differentially expressed genes at stage A1. Items = Codes of annotations, Items Details = description of annotations, Support = number of genes in input list with a given annotation, List size = number of genes in input list, Reference Support = number of genes in reference list with a given annotation, Reference size = number of genes in reference list, Hyp = Hypergeometric p-value, Hyp c = corrected Hypergeometric p-value (FDR), Genes = genes with given annotation in the input list. (XLS 40 KB)

Additional file 18: Table S16: Gene Ontology analysis (All annotations) of human homologs of differentially expressed genes at stage A2. Items = Codes of annotations, Items Details = description of annotations, Support = number of genes in input list with a given annotation, List size = number of genes in input list, Reference Support = number of genes in reference list with a given annotation, Reference size = number of genes in reference list, Hyp = Hypergeometric p-value, Hyp c = corrected Hypergeometric p-value (FDR), Genes = genes with given annotation in the input list. (XLS 33 KB)

Additional file 19: Table S17: Gene Ontology analysis of Biological Processes of human homologs of differentially expressed genes at stage A2. Items = Codes of annotations, Items Details = description of annotations, Support = number of genes in input list with a given annotation, List size = number of genes in input list, Reference Support = number of genes in reference list with a given annotation, Reference size = number of genes in reference list, Hyp = Hypergeometric p-value, Hyp c = corrected Hypergeometric p-value (FDR), Genes = genes with given annotation in the input list. (XLS 38 KB)

Additional file 20: Table S18: Gene Ontology analysis of Molecular Function of human homologs of differentially expressed genes at stage A2. Items = Codes of annotations, Items Details = description of annotations, Support = number of genes in input list with a given annotation, List size = number of genes in input list, Reference Support = number of genes in reference list with a given annotation, Reference size = number of genes in reference list, Hyp = Hypergeometric p-value, Hyp c = corrected Hypergeometric p-value (FDR), Genes = genes with given annotation in the input list. (XLS 120 KB)

Additional file 21: Table S19: Inter Pro analysis of human homologs of differentially expressed genes at stage A2. Items = Codes of annotations, Items Details = description of annotations, Support = number of genes in input list with a given annotation, List size = number of genes in input list, Reference Support = number of genes in reference list with a given annotation, Reference size = number of genes in reference list, Hyp = Hypergeometric p-value, Hyp c = corrected Hypergeometric p-value (FDR), Genes = genes with given annotation in the input list. (XLS 78 KB)

Additional file 22: Figure S2: Quantitative PCR of selected differentially expressed genes for fertile colonies throughout the entire blastogenic cycle of *Botryllus schlosseri*. Plots show upregulation of the selected genes for each gene are normalized to stage A1 showing fold change at each stage of the blastogenic cycle. Control reference gene is *ef-1*α. Error bars are from three biological and three technical replicates. (PDF 40 KB)

Additional file 23: Table S20: Gene Ontology analysis (All annotations) of human homologs of differentially expressed genes at stage B1. Items = Codes of annotations, Items Details = description of annotations, Support = number of genes in input list with a given annotation, List size = number of genes in input list, Reference Support = number of genes in reference list with a given annotation, Reference size = number of genes in reference list, Hyp = Hypergeometric p-value, Hyp c = corrected Hypergeometric p-value (FDR), Genes = genes with given annotation in the input list. (XLS 36 KB)

Additional file 24: Table S21: Gene Ontology analysis of Biological Processes of human homologs of differentially expressed genes at stage B1. Items = Codes of annotations, Items Details = description of annotations, Support = number of genes in input list with a given annotation, List size = number of genes in input list, Reference Support = number of genes in reference list with a given annotation, Reference size = number of genes in reference list, Hyp = Hypergeometric p-value, Hyp c = corrected Hypergeometric p-value (FDR), Genes = genes with given annotation in the input list. (XLS 48 KB)

Additional file 25: Table S22: Gene Ontology analysis of Molecular Function of human homologs of differentially expressed genes at stage B1. Items = Codes of annotations, Items Details = description of annotations, Support = number of genes in input list with a given annotation, List size = number of genes in input list, Reference Support = number of genes in reference list with a given annotation, Reference size = number of genes in reference list, Hyp = Hypergeometric p-value, Hyp c = corrected Hypergeometric p-value (FDR), Genes = genes with given annotation in the input list. (XLS 38 KB)

Additional file 26: Table S23: Inter Pro analysis of human homologs of differentially expressed genes at stage B1. Items = Codes of annotations, Items Details = description of annotations, Support = number of genes in input list with a given annotation, List size = number of genes in input list, Reference Support = number of genes in reference list with a given annotation, Reference size = number of genes in reference list, Hyp = Hypergeometric p-value, Hyp c = corrected Hypergeometric p-value (FDR), Genes = genes with given annotation in the input list. (XLS 40 KB)

Additional file 27: Table S24: Gene Ontology analysis (All annotations) of human homologs of differentially expressed genes at stage B2. Items = Codes of annotations, Items Details = description of annotations, Support = number of genes in input list with a given annotation, List size = number of genes in input list, Reference Support = number of genes in reference list with a given annotation, Reference size = number of genes in reference list, Hyp = Hypergeometric p-value, Hyp c = corrected Hypergeometric p-value (FDR), Genes = genes with given annotation in the input list. (XLS 36 KB)

Additional file 28: Table S25: Gene Ontology analysis of Biological Processes of human homologs of differentially expressed genes at stage B2. Items = Codes of annotations, Items Details = description of annotations, Support = number of genes in input list with a given annotation, List size = number of genes in input list, Reference Support = number of genes in reference list with a given annotation, Reference size = number of genes in reference list, Hyp = Hypergeometric p-value, Hyp c = corrected Hypergeometric p-value (FDR), Genes = genes with given annotation in the input list. (XLS 40 KB)

Additional file 29: Table S26: Gene Ontology analysis of Molecular Function of human homologs of differentially expressed genes at stage B2. Items = Codes of annotations, Items Details = description of annotations, Support = number of genes in input list with a given annotation, List size = number of genes in input list, Reference Support = number of genes in reference list with a given annotation, Reference size = number of genes in reference list, Hyp = Hypergeometric p-value, Hyp c = corrected Hypergeometric p-value (FDR), Genes = genes with given annotation in the input list. (XLS 28 KB)

Additional file 30: Table S27: Inter Pro analysis of human homologs of differentially expressed genes at stage B2. Items = Codes of annotations, Items Details = description of annotations, Support = number of genes in input list with a given annotation, List size = number of genes in input list, Reference Support = number of genes in reference list with a given annotation, Reference size = number of genes in reference list, Hyp = Hypergeometric p-value, Hyp c = corrected Hypergeometric p-value (FDR), Genes = genes with given annotation in the input list. (XLS 27 KB)

Additional file 31: Table S28: Gene Ontology analysis of Biological Processes of human homologs of differentially expressed genes at stage C1. Items = Codes of annotations, Items Details = description of annotations, Support = number of genes in input list with a given annotation, List size = number of genes in input list, Reference Support = number of genes in reference list with a given annotation, Reference size = number of genes in reference list, Hyp = Hypergeometric p-value, Hyp c = corrected Hypergeometric p-value (FDR), Genes = genes with given annotation in the input list. (XLS 34 KB)

Additional file 32: Table S29: Gene Ontology analysis of Molecular Function of human homologs of differentially expressed genes at stage C1. Items = Codes of annotations, Items Details = description of annotations, Support = number of genes in input list with a given annotation, List size = number of genes in input list, Reference Support = number of genes in reference list with a given annotation, Reference size = number of genes in reference list, Hyp = Hypergeometric p-value, Hyp c = corrected Hypergeometric p-value (FDR), Genes = genes with given annotation in the input list. (XLS 24 KB)

Additional file 33: Table S30: Inter Pro analysis of human homologs of differentially expressed genes at stage C1. Items = Codes of annotations, Items Details = description of annotations, Support = number of genes in input list with a given annotation, List size = number of genes in input list, Reference Support = number of genes in reference list with a given annotation, Reference size = number of genes in reference list, Hyp = Hypergeometric p-value, Hyp c = corrected Hypergeometric p-value (FDR), Genes = genes with given annotation in the input list. (XLS 36 KB)

Additional file 34: Table S31: Gene Ontology analysis (All annotations) of human homologs of differentially expressed genes at stage C2. Items = Codes of annotations, Items Details = description of annotations, Support = number of genes in input list with a given annotation, List size = number of genes in input list, Reference Support = number of genes in reference list with a given annotation, Reference size = number of genes in reference list, Hyp = Hypergeometric p-value, Hyp c = corrected Hypergeometric p-value (FDR), Genes = genes with given annotation in the input list. (XLS 26 KB)

Additional file 35: Table S32: Gene Ontology analysis of Biological Processes of human homologs of differentially expressed genes at stage C2. Items = Codes of annotations, Items Details = description of annotations, Support = number of genes in input list with a given annotation, List size = number of genes in input list, Reference Support = number of genes in reference list with a given annotation, Reference size = number of genes in reference list, Hyp = Hypergeometric p-value, Hyp c = corrected Hypergeometric p-value (FDR), Genes = genes with given annotation in the input list. (XLS 36 KB)

Additional file 36: Table S33: Gene Ontology analysis of Molecular Function of human homologs of differentially expressed genes at stage C2. Items = Codes of annotations, Items Details = description of annotations, Support = number of genes in input list with a given annotation, List size = number of genes in input list, Reference Support = number of genes in reference list with a given annotation, Reference size = number of genes in reference list, Hyp = Hypergeometric p-value, Hyp c = corrected Hypergeometric p-value (FDR), Genes = genes with given annotation in the input list. (XLS 38 KB)

Additional file 37: Table S34: Inter Pro analysis of human homologs of differentially expressed genes at stage C2. Items = Codes of annotations, Items Details = description of annotations, Support = number of genes in input list with a given annotation, List size = number of genes in input list, Reference Support = number of genes in reference list with a given annotation, Reference size = number of genes in reference list, Hyp = Hypergeometric p-value, Hyp c = corrected Hypergeometric p-value (FDR), Genes = genes with given annotation in the input list. (XLS 46 KB)

Additional file 38: Table S35: Gene Ontology analysis (All annotations) of human homologs of differentially expressed genes at stage D. Items = Codes of annotations, Items Details = description of annotations, Support = number of genes in input list with a given annotation, List size = number of genes in input list, Reference Support = number of genes in reference list with a given annotation, Reference size = number of genes in reference list, Hyp = Hypergeometric p-value, Hyp c = corrected Hypergeometric p-value (FDR), Genes = genes with given annotation in the input list. (XLS 36 KB)

Additional file 39: Table S36: Gene Ontology analysis of Biological Processes of human homologs of differentially expressed genes at stage D. Items = Codes of annotations, Items Details = description of annotations, Support = number of genes in input list with a given annotation, List size = number of genes in input list, Reference Support = number of genes in reference list with a given annotation, Reference size = number of genes in reference list, Hyp = Hypergeometric p-value, Hyp c = corrected Hypergeometric p-value (FDR), Genes = genes with given annotation in the input list. (XLS 42 KB)

Additional file 40: Table S37: Gene Ontology analysis of Molecular Function of human homologs of differentially expressed genes at stage D. Items = Codes of annotations, Items Details = description of annotations, Support = number of genes in input list with a given annotation, List size = number of genes in input list, Reference Support = number of genes in reference list with a given annotation, Reference size = number of genes in reference list, Hyp = Hypergeometric p-value, Hyp c = corrected Hypergeometric p-value (FDR), Genes = genes with given annotation in the input list. (XLS 36 KB)

Additional file 41: Table S38: Inter Pro analysis of human homologs of differentially expressed genes at stage D. Items = Codes of annotations, Items Details = description of annotations, Support = number of genes in input list with a given annotation, List size = number of genes in input list, Reference Support = number of genes in reference list with a given annotation, Reference size = number of genes in reference list, Hyp = Hypergeometric p-value, Hyp c = corrected Hypergeometric p-value (FDR), Genes = genes with given annotation in the input list. (XLS 42 KB)
