# Supplementary figures and images for: Analysis of the basal chordate Botryllus schlosseri reveals a set of genes associated with fertility
Source: BMC Genomics. 2014 Dec 26;15(1):1183. doi: 10.1186/1471-2164-15-1183 (PMC4523013; doi:10.1186/1471-2164-15-1183)

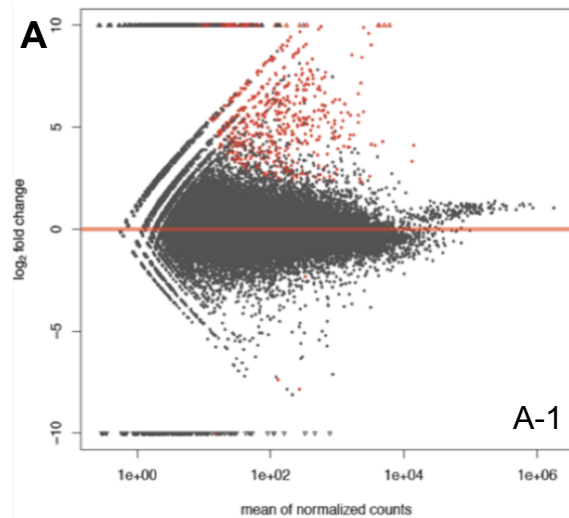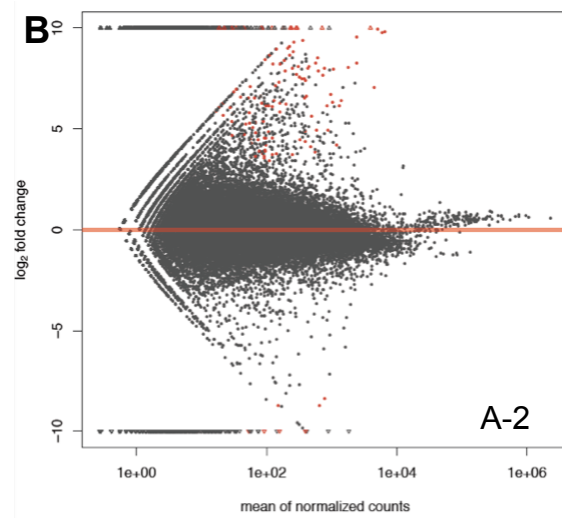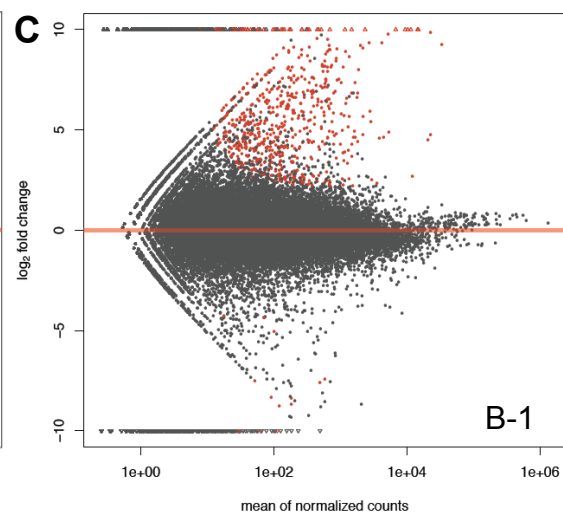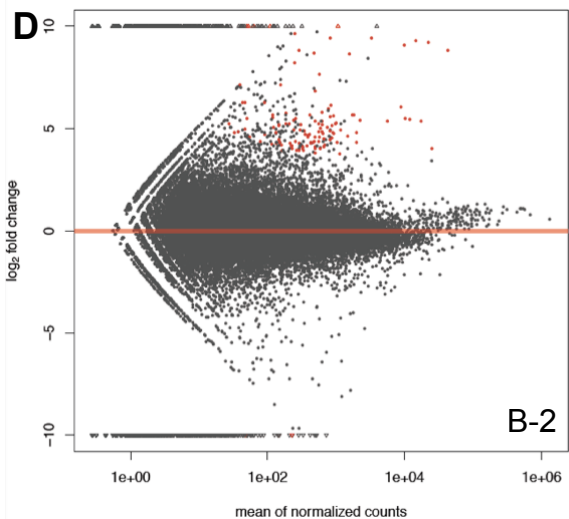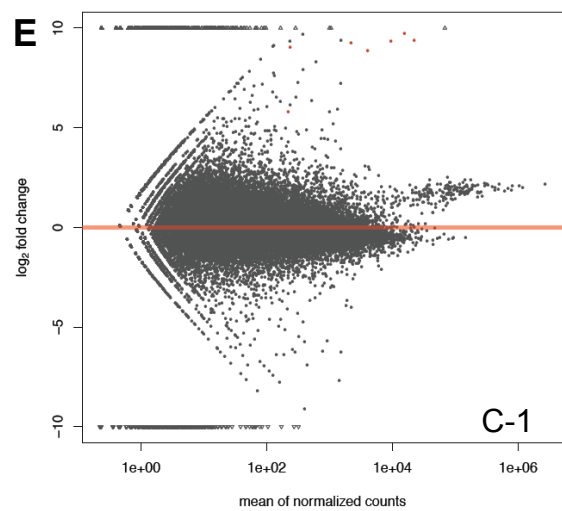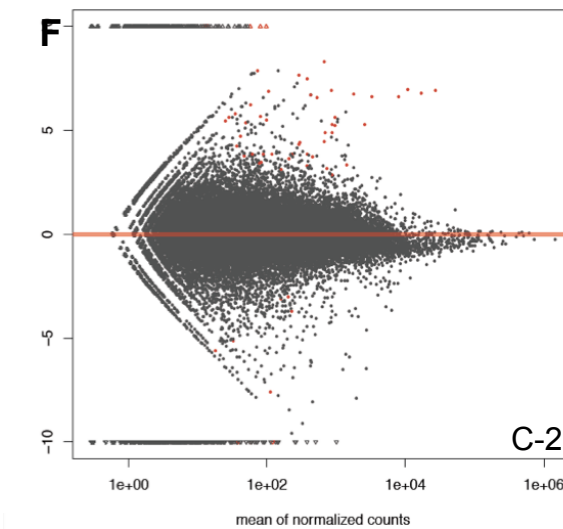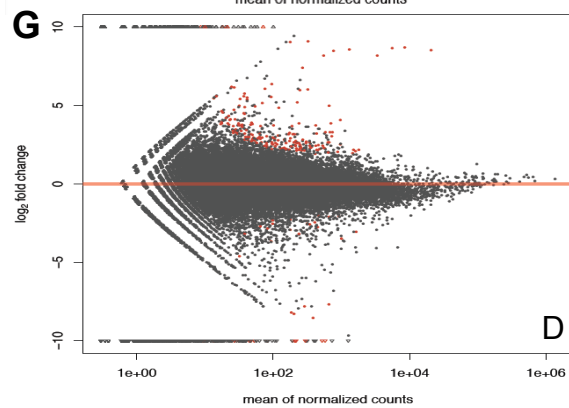

Supplement: Supplementary file 1 — Additional file 1: Figure S1. Scatter plots of Differentially Expressed genes throughout the entire blastogenic cycle of Botryllus schlosseri. Plot of normalized mean versus log2 fold change infertile versus fertile for each stage of the blastogenic cycle. Red circles indicate genes that are significant at a 10% false discovery rate (FDR). (PDF 615 KB) [file 12864_2014_7084_MOESM1_ESM.pdf]

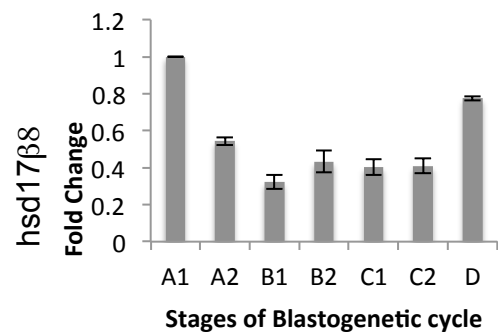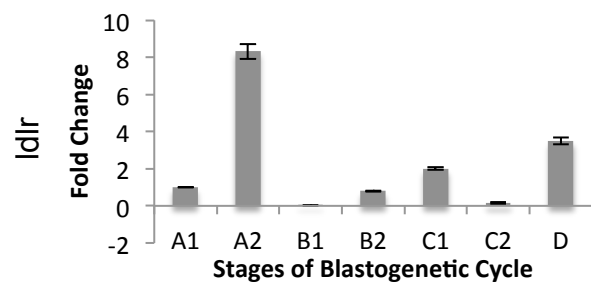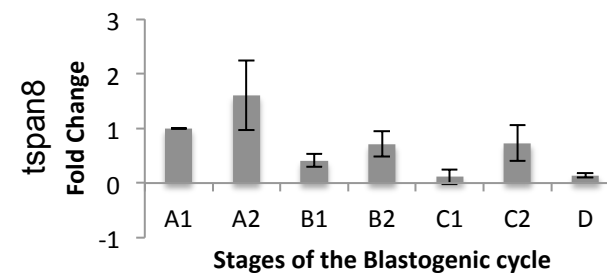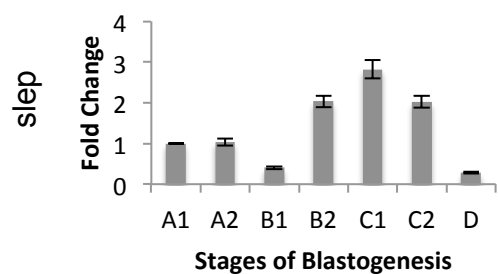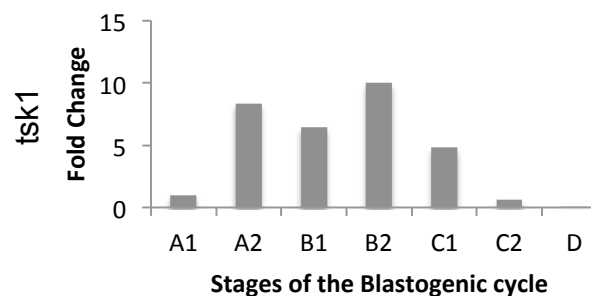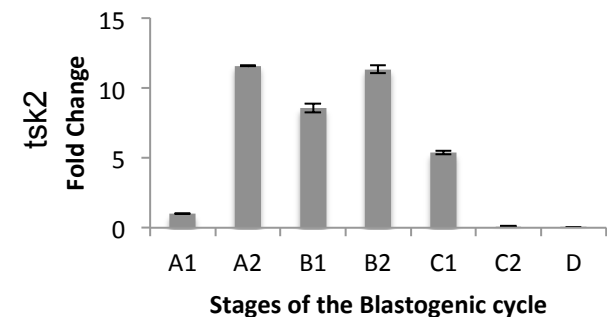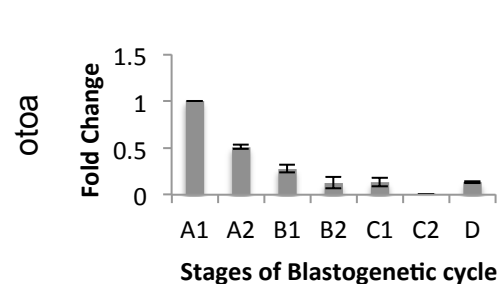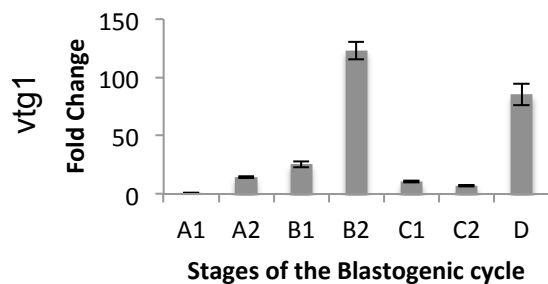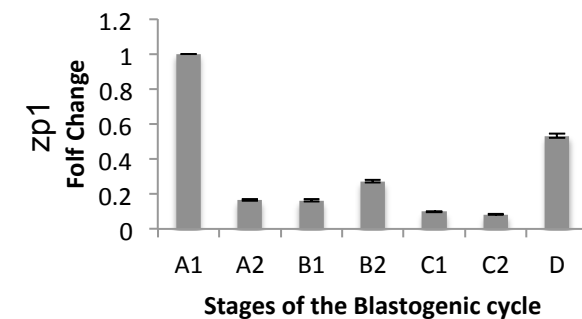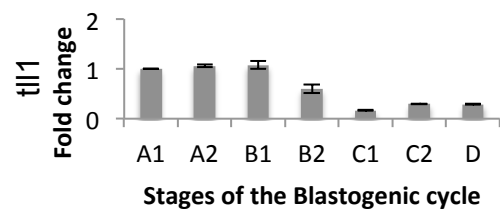

Supplement: Supplementary file 20 — Additional file 20: Table S18: Gene Ontology analysis of Molecular Function of human homologs of differentially expressed genes at stage A2. Items = Codes of annotations, Items Details = description of annotations, Support = number of genes in input list with a given annotation, List size = number of genes in input list, Reference Support = number of genes in reference list with a given annotation, Reference size = number of genes in reference list, Hyp = Hypergeometric p-value, Hyp c = corrected Hypergeometric p-value (FDR), Genes = genes with given annotation in the input list. (XLS 120 KB) [file 12864_2014_7084_MOESM20_ESM.xls]
